# 3D Anderson localization of classical scalar waves

**DOI:** 10.1126/sciadv.aed1319

**Published:** 2026-02-25

**Authors:** Fanambinana Delmotte, Thomas Brunet, Jacques Leng, John H. Page

**Affiliations:** ^1^ Univ. Bordeaux, CNRS, Bordeaux INP, I2M, UMR 5295, F-33400, Talence, France.; ^2^ Arts et Métiers Institute of Technology, CNRS, Bordeaux INP, I2M, UMR 5295, F-33400 Talence, France.; ^3^ Univ. Bordeaux, CNRS, Solvay, LOF, UMR 5258, F-33600 Pessac, France.; ^4^ Department of Physics, University of Manitoba, Winnipeg, Manitoba, R3T 2N2, Canada.

## Abstract

Anderson localization, first predicted in 1958, is one of the most fascinating and remarkable wave phenomena. The experimental search for this halt of diffusive transport in three-dimensional (3D) disordered systems, which is still very actively pursued for classical waves (light or sound), has never been demonstrated for the fundamental case of scalar waves. Inspired by recent advances in 3D metamaterials, we show that a locally resonant ultrasonic metafluid consisting of a suspension of soft metallic beads is the innovation needed for 3D Anderson localization of scalar acoustic waves to be definitively observed. By reporting two independent sets of time- and position-resolved ultrasonic experiments, we present clear evidence of Anderson transitions between diffusion and localization, accurately determining the mobility edges and the localization phase diagram.

## Teaser

In a locally resonant metafluid with optimal disorder, strong scattering completely blocks the diffusive transport of sound waves.

## INTRODUCTION

Anderson localization (*1*) originates from the interferences between multiple scattering paths and may occur for any type of wave (quantum or classical), thereby involving a wealth of wave physics, particularly in three-dimensional (3D) systems where there is a real transition between diffusive and localized behavior. Although already observed for quantum particles (*2*–*4*), experimental searches for 3D Anderson localization of classical waves (*5*, *6*) are still challenging because they require strongly disordered systems with severe constraints (strong scattering, very low absorption, etc.). In optics, several strongly scattering powders (*7*, *8*) were investigated in the hope of observing localization of light in three dimensions, but all have proved unsuccessful due to confounding effects (absorption, fluorescence, and near-field longitudinal couplings) (*9*–*16*). More recently, a numerical study has proposed 3D localization of electromagnetic waves in randomly packed, overlapping metallic spheres (*17*), but this also has yet to be confirmed experimentally. By contrast, the first convincing experimental evidence for 3D localization of classical waves was found for ultrasound in 3D mesoglasses (*18*–*22*), which are an experimental realization for classical waves of the “tight-binding” model often used to study electron localization (*1*). However, these experiments all involved vector waves, either in optics (two transverse polarizations) or in ultrasonics (mesoglasses are solids that support one longitudinal and two transverse polarizations), and, consequently, experimental evidence for 3D Anderson localization of scalar classical waves has remained elusive until now.

For decades, experimentalists have realized that success in demonstrating scalar wave localization for the first time would be a major achievement that would advance the state of the art in wave localization research. One reason is that, for classical waves, scalar wave localization corresponds most closely to the original theory of localization by Anderson himself, who considered the localization of electrons that are described by scalar wave functions. However, for electrons, interaction effects can mask experimental investigations of localization, so that the ideal way of studying the most fundamental aspects of localization is to use scalar acoustic waves in a fluid-like linear medium that supports only pressure waves (no polarization effects and no wave interaction effects). Furthermore, since most theoretical work on localization has considered only scalar waves, these theories are the basis of much of our current understanding of Anderson localization, and a fully meaningful validation of these scalar wave theories requires experiments on scalar wave materials. Hence, the experimental search for 3D Anderson localization of scalar classical waves has become like the pursuit of the holy grail, since it is the simplest case that enables the fundamentals of localization to be probed and tested most clearly without complications due to polarization effects or interactions.

Reaching a localization transition requires very strong scattering, which means very small values of $k\ell_{s}$, with k being the effective wave number in the medium and $\ell_{s}$ the scattering mean free path. To minimize the product $k\ell_{s}$, taking advantage of scattering resonances can prove effective in lowering $\ell_{s}$ since they can lead to the rapid extinction of waves traveling through a fully random medium (*23*). In acoustics, the first studies involved suspensions of commercially available spherical scatterers, e.g., suspensions of (hard) glass spheres in water, for which the scattering was too weak despite a strong tortuosity resonance at high particle concentrations (*24*, *25*), and suspensions of (soft) plastic spheres, which exhibited stronger resonant scattering but prohibitively high absorption (*26*). Notably, the craze for acoustic metamaterials (*27*, *28*), which began in 2000 with the achievement of the first locally resonant sonic materials (*29*), paved the way for the production of various resonant particles, as demonstrated a few years later using soft matter techniques (*30*), which showed that remarkable scattering media could be synthesized with well-controlled customized properties. In particular, the simultaneous monopolar and dipolar Mie-type resonances of soft porous silicone-rubber microbeads embedded in a yield-stress fluid enabled the first acoustic metafluids with a negative acoustic index to be realized (*31*). Unfortunately, the experimental search for 3D Anderson localization with these soft resonant particles is illusory due to their high intrinsic absorption (*32*). Similarly, although they are well known for a long time to exhibit giant monopolar resonances (*33*), air bubbles are not good candidates either, because of the excessive absorptive losses they induce (*34*). Alternatively, Tallon *et al.* have recently taken advantage of multipolar resonances of very weakly absorbent (liquid) fluorinated oil droplets embedded in a weakly absorbent yield-stress fluid to study the strong impact of scattering resonances on all the key transport parameters of classical waves in disordered media (*35*). However, even for an optimal concentration of droplets, the scattering was still too weak to reach a localized regime (*36*). Hence, 3D Anderson localization of scalar acoustic waves has never been convincingly demonstrated to date, possibly implying that scalar wave localization in 3D disordered materials might even be unattainable, as suggested more generally for randomly dispersed resonant scatterers by John many years ago (*37*).

In the present work, we demonstrate unambiguously that scalar waves may indeed undergo 3D Anderson localization. To do so, we design and engineer a specific 3D metafluid that exhibits very strong resonances and low absorption, and we deploy a precise methodology that validates a transition between diffusive and localized regimes. Notably, two parameters affect the mobility edges in between which 3D Anderson localization emerges, and we establish a frequency-concentration map of scalar wave localization. This phase diagram obtained in a 3D disordered medium encapsulates a major breakthrough that promises a rich research playground for the soon coming, next generations of 3D metafluids with tailored spatial correlations.

## RESULTS

### A strongly scattering 3D metafluid with low absorption

**Fig. 1.**
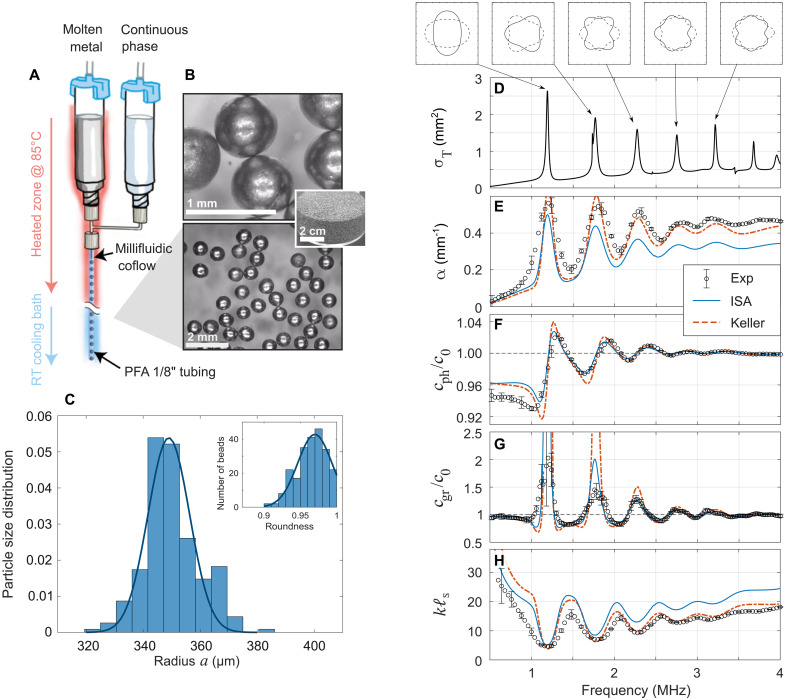
Metafluid with soft resonant metallic precision microbeads. (**A**) Microfluidic setup used for fabricating precision beads. (**B**) Pictures of these beads at different scales. (**C**) Particle size distribution obtained by optical methods for beads with average radius 〈*a*〉 = 349 μm (±2.2%). (**D**) Theoretical scattering cross section $\sigma_{T}$ of a single bead, with the multipolar nature of the bead resonances illustrated by the diagrams above the graph. In figures (**E**) to (**H**), circles represent the measured attenuation α, normalized phase velocity, normalized group velocity, and $k\ell_{s}$ product, respectively, for ϕ=20%. The data are averaged measurements for different propagation distances ranging from 3 to 8 mm in 1-mm steps, and the error bars are the standard deviations. Theoretical calculations are based on the ISA (solid blue line) and the Keller model (dashed orange curve). Exp is an abbreviation for experimental data.

As a key ingredient to alter the transport of scalar waves, we developed a sophisticated method for randomly seeding a passive, nonabsorbent aqueous gel with spherical inclusions (low-loss, Mie-type resonators) that display exceptional scattering strength (see Materials and Methods). These resonators were made of a fusible alloy of bismuth, lead, indium, tin, and cadmium (Indalloy 117 by Indium Corp., USA), a soft metal that is a solid at room temperature with a low shear modulus. It exhibits a low melting point (≈47°C), which allows it to be formed into highly calibrated precision microbeads using microfluidics (Fig. 1A) (*38*). Not only are the resonators near-perfect spheres (radius, ~350 μm; dispersity, ~2%) (Fig. 1, B and C), but we could also produce very large quantities of them, of the order of 0.1 kg/hour (i.e., ~10^5^ particles/hour), which represents a tour de force in the field of microfluidic-assisted production of functional particles (*39*, *40*). It is also a prerequisite for enabling ultrasonic experiments to be carried out on large sample volumes and for a wide range of resonator concentrations.

The sharp acoustic, Mie-type resonances of a single bead were calculated and appear as narrow peaks in the scattering cross section ($\sigma_{T}$, Fig. 1D; see also Materials and Methods). These resonances have a massive impact on the scattering strength and therefore on acoustic wave propagation in the suspensions, as determined by measuring the pulsed, ensemble-averaged transmitted wave field ⟨ψ(t)⟩, which travels ballistically straight through a sample in the forward direction. This wave pulse carries information on the scattering mean free path $\ell_{s}$, as well as the phase $c_{\text{ph}}$ and group $c_{\text{gr}}$ velocities (*25*), thereby enabling the scattering strength at angular frequency $\omega ,k\ell_{s}=\left(\omega /c_{\text{ph}}\right)\ell_{s}$, to be directly measured. The measurements were carried out for a wide range of bead volume fractions, ϕ∈[2.5−40]%, with representative data at the intermediate concentration of ϕ=20% being shown in Fig. 1 (E to H). At the resonances, the phase velocity (Fig. 1F) varies rapidly with frequency, and the group velocity (Fig. 1G) shows rapid growth toward values larger than $c_{0}$, the sound speed in the pure gel. Crucially, the attenuation coefficient α (Fig. 1E) exhibits large maxima, leading to notable minima in $k\ell_{s}$ (Fig. 1H).

These data are compared with predictions of the independent scattering approximation (ISA) (*23*) and with the Keller model (*41*) (Fig. 1). Among the various models that incorporate higher-order corrections, the Keller model gives the best agreement by accounting for the finite sizes of the microbeads. Overall, the measurement of the coherent average wave reveals the key role of the local resonances on the properties of the suspensions. It is especially true for the sharp decrease of the scattering mean free path $\ell_{s}$ at some frequencies such as near the quadrupolar resonance (f= 1.20 MHz) where $\ell_{s}/\lambda =0.73\pm 0.06$ and $k\ell_{s}=4.6\pm 0.4$, indicating that, here, the waves are very strongly scattered and that this represents the best frequency range for seeking direct evidence of Anderson localization.

### Confinement of the scattered intensity

**Fig. 2.**
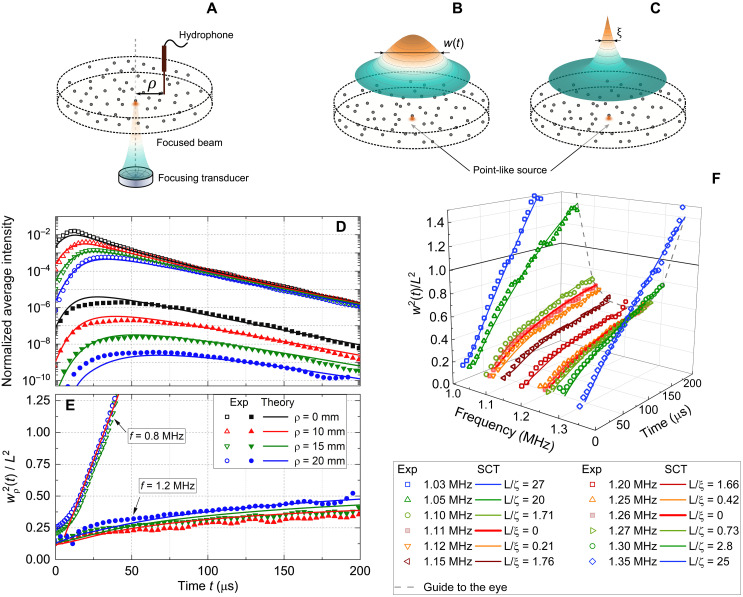
Transverse spreading and confinement of the multiply scattered intensity. (**A**) Schematic diagram of the experimental setup, showing the incident point-like beam and position-resolved detection of the transmitted multiply scattered waves. (**B**) Schematic illustration of the behavior in the diffuse regime: The transverse spatial profile of the transmitted intensity (the TOF profile) spreads without limit. (**C**) Schematic illustration for the localized regime: The profile is confined to a narrow region opposite the source. (**D** and **E**) Representative results for ϕ=20% of the measured and theoretically predicted TOF and $w_{\rho }^{2}\left(t\right)$ in the diffuse (f = 0.8 MHz, open symbols) and localized (f = 1.2 MHz, solid symbols) regimes, respectively. Error bars for the experimental data (not shown) are smaller than or comparable to the size of the symbols; for the TOFs, they represent the standard error of measurements over different disorder configurations that were probed using 441 independent source positions, while for $w_{\rho }^{2}\left(t\right)$, they were obtained by error propagation. In (D), the comparison of the data with the diffusion approximation yields $D_{B}=1.5\pm 0.1$ mm^2^/μs, whereas in (E), the best SCT fit gives L/ξ = 1.7 ± 0.3. (**F**) Time dependence of the width squared at ρ = 20 mm for various frequencies encompassing the mobility gap between the lower and upper mobility edges at 1.11 and 1.26 MHz. Error bars are calculated as in (E).

The confinement of the scattered intensity is an unambiguous signature of localization and is arguably its essence. We therefore investigated the transport behavior of the multiply scattered acoustic waves, $\delta \psi =\psi_{\text{tot}}-\langle \psi \rangle$, using a method that directly measures the transverse confinement of the intensity in a plane perpendicular to the incident beam (*18*, *42*). Experimentally, the transmission of the dynamic wave field from a point-like pulsed source is measured on the opposite side of a slab-shaped sample using a needle hydrophone (Fig. 2A). By digitally filtering these signals, the time-, position-, and frequency-resolved transmitted field ${\delta \psi }_{\omega }\left(\rho ,t\right)$ is recorded, with ρ the transverse position along the sample surface. Of prime interest, the time-of-flight (TOF) profile $I_{\omega }\left(\rho ,t\right)$ is obtained by configurationally averaging the envelope of the square of the field and normalizing it by the corresponding input signal. The intensity ratio $I_{\omega }\left(\rho ,t\right)/I_{\omega }\left(\rho =0,t\right)$ is independent of boundary conditions as well as absorption (*18*, *42*), which has plagued some previous attempts to investigate Anderson localization (*7*, *8*). In the diffusive regime, this intensity ratio spreads laterally without limit, and its transverse width squared $w^{2}\left(t\right)=-\rho^{2}/\text{ln}\left[I_{\omega }\left(\rho ,t\right)/I_{\omega }\left(0,t\right)\right]=4D_{B}t$ increases linearly with time, enabling the measurement of the Boltzmann diffusion coefficient $D_{B}$ (*24*). However, in the localization regime, the lateral expansion of the intensity ratio becomes confined (compare Fig. 2, B and C), and, consequently, the transverse width saturates to a constant value at long times.

These dramatically different behaviors are experimentally demonstrated by representative data for $I_{\omega }\left(\rho ,t\right)$ and $w_{\rho }^{2}\left(t\right)$ at ϕ=20% (Fig. 2, D and E) for two frequencies, below (*f* = 0.80 MHz) and at resonance (f=1.20 MHz) where the scattering is maximized. At the lower frequency, the linear increase of the width squared from *w* = 0 at *t* = 0 shows simple diffusive behavior ($w^{2}=4D_{B}t$), while at resonance, the width squared has clearly bent over and is approaching a constant at long times. Also, the TOF profiles for different ρ exhibit different long-time trends, either approaching each other in the diffusive case or remaining well separated when localization occurs. Furthermore, in and near the localization regime, the TOF profiles become non-Gaussian, and, consequently, the width squared as defined above acquires a weak ρ dependence.

To further quantify the behavior observed near the Anderson localization transitions and to delineate its mobility edges, we compared the data with theoretical predictions of the Self-Consistent Theory (SCT) of localization, which accounts for the renormalization of the diffusion coefficient by interferences due to Anderson localization and the resulting spatial dependence in finite samples of the diffusion coefficient $D\left(\vec{r}\right)$ (*43*, *44*). Simultaneous fitting of SCT predictions to experimental data for $I_{\omega }\left(\rho ,t\right)$ and $w_{\rho }^{2}\left(t\right)$ (see Materials and Methods for details) enabled us to determine the localization length ξ or correlation length ζ of fluctuations in the diffusive regime, whose respective magnitudes identify the proximity to a mobility edge at an Anderson transition. The agreement between theory and experiments is very good at all frequencies, and, at resonance (Fig. 2, D and E, solid symbols and curves), the localization length ξ is significantly less than the sample thickness *L*, thus confirming that the sample is well within a localization regime. The emergence of localization is best seen in Fig. 2F, as the ultrasonic frequency (i.e, scattering strength) is varied through the Anderson transitions in this sample: The width squared progressively bends over more and more as the localization regime is approached from below and then increases again at long times for frequencies above the most localized frequency at 1.2 MHz. Two mobility edges are thus revealed at *f* = 1.11 and 1.26 MHz (within 0.6%), where the divergence of ξ and ζ (L/ξ=L/ζ=0) brackets the Anderson mobility gap (red symbols and curves in Fig. 2F).

### Speckle pattern intensity statistics

**Fig. 3.**
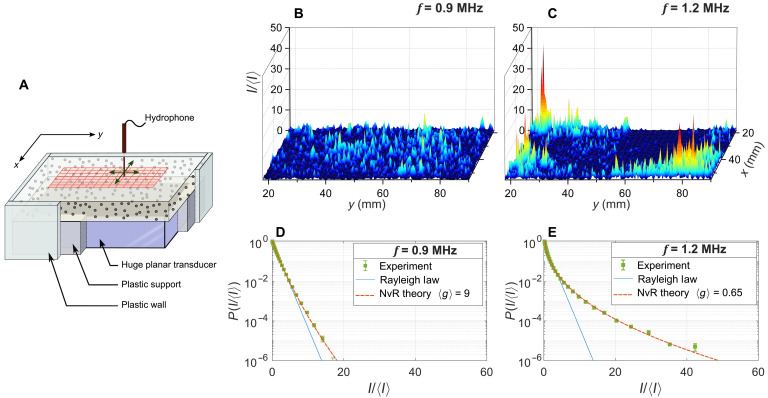
Statistical approach to localization. (**A**) Diagram of the experimental setup for point-by-point measurements of the transmitted field, from which speckle patterns and the statistical analysis of the intensity fluctuations were determined. The diagram shows the hydrophone at the top, the rectangular transducer at the bottom, and the sample in the middle. (**B** and **C**) Speckle patterns for ϕ=20% at a frequency below (0.9 MHz) and in (1.2 MHz) the localized regime, respectively. (**D** and **E**) The corresponding probability density functions at these two frequencies. The experimental error bars represent the standard error of measurements for four statistically equivalent samples.

**Fig. 4.**
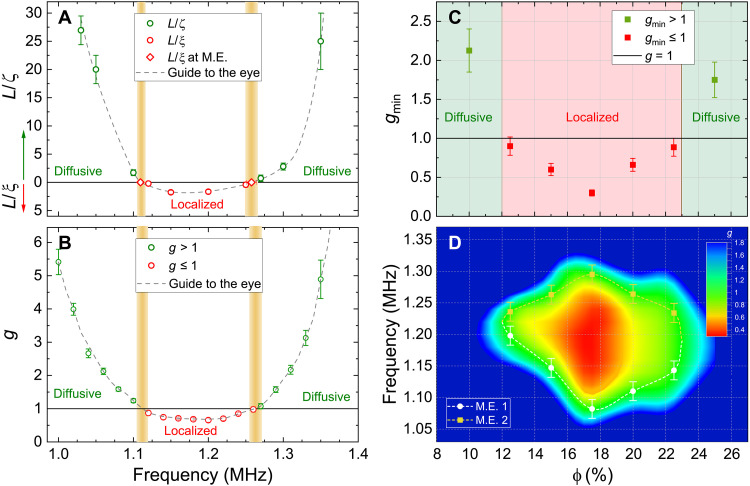
Anderson mobility gap. (**A** and **B**) Evolution of the ratio L/ξ and L/ζ (top) and the dimensionless conductance g (bottom) as a function of frequency for ϕ=20%. Mobility edges (M.E.) occur at 1.11 and 1.26 MHz when L/ξ=L/ζ = 0 and g=1; they are indicated by the blurred vertical bars whose width denotes their 0.6% uncertainty. The agreement between the location of these mobility edges by the two independent measurements is excellent. (**C**) The minimum values of the dimensionless conductance for each concentration investigated. (**D**) Volume fraction-frequency cartography highlights the mobility edges (symbols), determined from g measurements (color coded). Together, (C) and (D) indicate that the bead volume fractions for which Anderson localization occurs extend only from 12 to 23%, with an uncertainty of ~0.5%. The error bars in (A) and (B) indicate the standard deviations of the most probable values, as described in the Materials and Methods; these uncertainties were used to estimate the error bars in (C) and (D).

Statistical analysis of the speckle pattern provides complementary evidence of Anderson localization (*18*, *45*). Experimentally, we ensured a broad incident beam by placing the sample directly on top of a huge planar rectangular transducer and mapped the transmitted field using a hydrophone (Fig. 3A). From these data, the transmitted intensity, normalized by its average value, $I\left(x,y\right)/{\left\langle I\left(x,y\right)\right\rangle }_{x,y}$, was determined and plotted as 3D color maps, shown for two frequencies in Fig. 3 (B and C). These speckle patterns reveal two very different fluctuation typologies, with overlapping low-intensity peaks below resonance (*f* = 0.90 MHz; Fig. 3B) but with high-intensity peaks in some regions and low intensities elsewhere at resonance (*f* = 1.20 MHz; Fig. 3C). This behavior is quantified by the corresponding probability distribution functions, P(I/⟨I⟩), which show small deviations from Rayleigh statistics (Fig. 3D, dashed blue line) below resonance but huge deviations and large probabilities of very large intensities at resonance (Fig. 3E). The data are well described using predictions in (*46*) [Nieuwenhuizen and van Rossum theory (NvR) theory, orange line] from which we extracted the dimensionless conductance g, a universal scaling parameter of localization, and obtained g < 1 (=0.65) at resonance, as expected in the localization regime (*47*), but g > 1 (=9) at the lower frequency, typical of a diffusive regime. The frequency dependence of g was also investigated (Fig. 4B) and revealed clear evidence of a mobility gap; notably, it perfectly matches the one determined independently using the transverse confinement measurements of L/ξ and L/ζ (replotted in Fig. 4A).

In addition, we took advantage of the versatility of our inclusion-based metafluid to vary systematically the resonator concentration, and monitored its impact on the mobility gap. The measured minimal conductance g for each ϕ reveals that Anderson localization is observed only over a finite range of concentrations at the intermediate volume fractions ϕ∈[12−23]% (with an uncertainty of ~0.5%; Fig. 4C). Furthermore, by establishing the frequency-concentration cartography of localization for this system (Fig. 4D), i.e., the localization phase diagram, we show that specific conditions are required to observe it, and we determined the deepest possible localized state. Whereas scattering is too weak at low concentrations to achieve localization, scattering is renormalized at high concentrations so that the effective medium around each scatterer becomes similar to the scatterer itself, thereby weakening the scattering strength. This localization phase diagram encapsulates this important experimental demonstration that the particle concentration in strongly scattering resonant suspensions must be limited to an intermediate range for Anderson localization of classical waves to occur. Although the exact range remains system dependent, the phenomenon is expected to be universal for systems consisting of strong resonators dispersed in a continuous medium.

## DISCUSSION

Anderson localization, predicted over half a century ago, has never been convincingly demonstrated previously for scalar waves in 3D disordered systems. Here, we not only provide an unambiguous experimental proof of localization for scalar acoustic waves in a carefully designed, fairly generic and customizable 3D metafluid, but we also demonstrate that this phenomenon is restricted to a frequency bandwidth close to resonance and to a specific, intermediate concentration range of resonators. This generic behavior is expected to be universal, even though the specific frequency-concentration range is system sensitive (resonator material and size).

Our observation that localization occurs only at an intermediate concentration of particles may be considered especially interesting in view of recent numerical predictions for the localization of electromagnetic waves. These simulations were performed on 3D random aggregates of overlapping metallic spheres and showed that high volume fractions of ~50% (or greater) were needed for localization to occur. However, in this system, since electromagnetic waves cannot penetrate inside the spheres (the skin depth is much less than the radius of the spheres), propagation occurs only in the air channels around the spheres and is most hindered by the narrowest gaps between them. Hence, the strongest scattering only occurs at high particle concentrations where the tortuosity of the multiple scattering paths is greatest and the gaps between spheres are smallest (subwavelength). This situation is analogous to, but the inverse of, the transport of elastic waves through mesoglasses, where the waves travel only through the network of spheres, since acoustic and elastic waves cannot exist in the evacuated pore space surrounding the solid network. For these mesoglasses where Anderson localization has been observed experimentally, the sphere concentration was also high and was ~55%. Hence, the nature of wave propagation is very different in these two tortuous systems from that of wave transport in the medium investigated in this paper, and explains why different concentration ranges are needed for localization to occur. Because our case of wave transport through a continuous medium in which strongly scattering resonant particles are embedded is the archetypical medium most frequently considered for localization of classical waves, our results for Anderson localization of scalar waves and the associated experimental phase diagram may be considered a major accomplishment.

Our breakthrough has been made possible by innovations in microfluidic particle synthesis, and points the way forward to future progress in fabricating samples with unique wave fields and structure factors as a result of exquisite control over resonator properties such as their composition, size, shape, position, and concentration. In particular, the potential for precise programming of the resonator locations, most likely by robotic micropositioning (*48*), will open up unprecedented configurations for the study of scalar wave transport in complex correlated disordered media (*49*), ranging from short-range near-neighbor correlations (e.g., pair correlations and cluster correlations) all the way to long-range correlations with bandgap effects that can facilitate the occurrence of 3D localization [see, e.g., theoretical and numerical predictions involving bandgaps in optics; (*37*, *50*–*52*)]. The fabrication of such correlated structures for scalar waves will enable many opportunities for advancing our understanding of wave physics involving and transcending localization.

## MATERIALS AND METHODS

### Precision microbead fabrication

As illustrated in Fig. 1A, the microfluidic setup developed to produce the soft metallic microbeads (with longitudinal and transverse phase velocities $c_{L}=2.37\;\text{mm}\;\mu s^{-1}\;\mathrm{and}\;c_{T}=1.03\;\text{mm}\;\mu s^{-1}$, $\rho =9.16\;g\;cm^{-3}$, and attenuation α too weak to be measured) consists of a coflow geometry where a pressurized cartridge of heated alloy permits the fragmentation of the liquid metal into calibrated droplets when put in contact with a preheated continuous phase that contains water, glycerol, polyvinyl alcohol (PVA) as a stabilizer, and some HCl to prevent surface oxidation of the alloy. Any cold spot is redhibitory and may block the fate of the segmented flow; care has to be taken as to the precise temperature regulation at any point of the device. In addition, the injection nozzle (a metallic needle) has to be surface treated, here by fluorination, to prevent local fouling, which tends to block the flow. While the still-liquid drops are flowing in the outlet tubing, the latter is immersed in a cold bath that ensures the quenching of the particles in order to turn them into solid particles, which are then collected in a bath under gentle stirring. The particles are then dispersed at the required volume fraction in a yield-stress fluid (with properties $c_{0}=1.495\;\text{mm}\;\mu s^{-1},\alpha_{0}=5\times 10^{-5}\;\text{MH}z^{-2}\;mm^{-1}$, and $\rho_{0}=1.005\;g\;cm^{-3}$) that prevents their sedimentation. This gel is prepared according to the following protocol: 1 wt % Carbopol ETD 2050 powder is dissolved in deionized water at 50°C under stirring for 1 hour. Since the resulting mixture is acidic (pH ≈ 3), sodium hydroxide (NaOH) is added after cooling to room temperature to adjust the pH to 7 and induce gelation. The last step is to place the gel in a vacuum chamber to remove unwanted bubbles.

This optimization of the microfluidic setup has permitted a high-throughput production of precision microbeads with well-controlled diameter. For example, a final 20 ml of gel sample loaded with 20% volume fraction of the resonating beads can be produced in a matter of a couple of hours, which is a sufficiently rapid rate of production to achieve the large number of beads needed for meaningful Anderson localization experiments.

### Coherent ballistic wave pulse

The coherent ballistic average wave pulse, ⟨ψ(t)⟩, was measured using a pair of large-diameter transducers (emitter/receiver) in direct contact with each sample, which was placed between the two transducers (*35*). We used the ergodicity of the random suspension to replace configurational averaging by spatial averaging of the wave field over the wide surface of the transducer. Additional ensemble averaging was performed, if needed, by repeating the measurements on samples from the same mixture but with different configurations of the scatterers, and also by performing measurements on samples with different thicknesses ranging from 3 to 8 mm. Hence, a meaningful ensemble average was obtained, and the average wave pulse could be accurately extracted from the total transmitted field. This average wave propagates as if it were in a homogeneous medium with effective wave number k from which the basic scattering properties of the medium were obtained. Comparing the phase and the amplitude of the transmitted coherent wave with a reference signal that traveled though the pure gel (subscript 0), we can extract all the effective parameters

$$\alpha \left(\omega \right)=-\frac{1}{d}\text{ln}\frac{|\text{FFT}\left\{\langle \psi \rangle \left(t,d\right)\right\}|}{|\text{FFT}\left\{\psi_{0}\left(t,d\right)\right\}|}+\alpha_{0}$$

$$\frac{1}{c_{\text{ph}}\left(\omega \right)}=\frac{\varphi \left(\omega ,d\right)-\varphi_{0}\;\left(\omega ,d\right)}{\omega d}+\frac{1}{c_{0}}$$

$$\frac{1}{c_{\text{gr}}\left(\omega \right)}=\frac{t_{\text{pk}}\;\left(\omega \right)-t_{\text{pk},0}\;\left(\omega \right)}{d}+\frac{1}{c_{0}}$$

Here, FFT represents fast Fourier transform, d is the propagation distance, α is the attenuation coefficient, φ is the (cumulative) phase of the signal, and $t_{\text{pk}}$ is the group time. The scattering mean free path $\ell_{s}$ is directly related to the attenuation coefficient α of the coherent wave since $2\alpha =1/\ell_{\text{ext}}=1/\ell_{s}+1/\ell_{a}$, where $\ell_{\text{ext}}$ is the extension length and $\ell_{a}$ the absorption length. At the frequencies of interest where the scattering is strong, we estimated the absorption length, on the basis of independent measurements for the multiply scattered waves, to be roughly $100\times \ell_{\text{ext}}$, so that $\ell_{s}=\ell_{\text{ext}}$ to an excellent approximation ($\ell_{a}\gg \ell_{s}$). Hence, the scattering strength at angular frequency $\omega ,k\ell_{s}=\left(\omega /c_{\text{ph}}\right)\ell_{s}=\left(\omega /c_{\text{ph}}\right)/2\alpha ,$ was also directly obtained from these experiments.

The methods used to interpret these data involved predictions based on the ISA (*23*) and the Keller model (*41*) (Fig. 1). The ISA results in the simplest model that predicts the substantial impact of resonances on the transport of the ballistic pulse (blue curves in Fig. 1). In this approximation, the effective wave number k is calculated from the scattering properties of a single scatterer

$$k^{2}={\left(\frac{\omega }{c_{\text{ph}}}+i\alpha \right)}^{2}=k_{0}^{2}+4\pi \int_{a}\eta_{a}f_{a}\left(0\right)\mathrm{da}$$

Here, η is the number of beads per unit volume, *f*(0) is the forward scattering amplitude of a single bead, and i represents $\sqrt{-1}$. To account for data at intermediate concentrations of scatterers, it was necessary to invoke higher order corrections, such as positional correlations due to the finite size of the beads that are included in the Keller model via the pair correlation function $g_{\text{pr}}\left(r\right)$. In this model, the wave number is expressed as

$$\begin{array}{cc} k^{2} & =k_{0}^{2}+4\pi \int_{a}f_{a}\left(0\right)\eta_{a}\mathrm{da}\\ & -{\left(4\pi \right)}^{2}\int_{a}{\left[f_{a}\left(0\right)\eta_{a}\right]}^{2}\int_{r}\frac{\left[1-g_{\text{pr}}\left(r\right)\right]\text{sin}\left(\mathrm{kr}\right)}{k}\text{exp}\left(ik_{0}r\right)\text{drda} \end{array}$$

which shows how the impenetrable particle condition known as the “Hole Correction” is incorporated into the model [$g_{\text{pr}}\left(r\right)=1$ if r>2a and 0 elsewhere for the case considered here].

### Multiply scattered transmitted intensity

The sample, source, and detector used to measure the transmitted multiply scattered waves were immersed in a large water tank, since water is a convenient medium to facilitate the propagation of ultrasonic waves to and from the sample. Specifically, the experimental setup consists of (i) the sample contained in a large diameter cylindrical cell (∅ ∅>10L) having a lateral wall made from polyvinyl chloride and front and back walls made from thin sheets of polycarbonate (with a thickness of 0.5 mm); (ii) a pulsed, broadband focusing transducer (with a central frequency of 1 MHz) for emission, mounted to a conical aperture to suppress secondary side lobes and positioned on one side of the sample; and (iii) a subwavelength hydrophone for reception located at the opposite side of the sample. Both the hydrophone and the sample were moved parallel to the front and back surfaces of the sample by translation stages, allowing the transmitted field to be measured over 441 independent source positions to scan different disorder configurations and, for each source position, five transverse detector distances (ρ = 0, 5, 10, 15, and 20 mm). For each sample and hydrophone position, the measurement of the total transmitted wave field was repeated many times and averaged to reduce background noise. For each acquisition, we suppress the coherent ballistic wave pulse ⟨ψ(ρ,t)⟩ to extract the position-and-time resolved multiply scattered waves $\delta \psi \left(\rho ,t\right)=\psi_{\text{total}}\left(\rho ,t\right)-\left\langle \psi \left(\rho ,t\right)\right\rangle$. Frequency-dependent TOF profiles were obtained by digitally filtering the signals and taking the square of the envelope. The standard deviation of the Gaussian filter was equal to ∆f = 20 kHz. Note that the method to characterize the expansion (or not) of the multiply scattered halo in terms of the width squared results in an absorption-free measure, since absorption enters as a multiplicative factor $\text{exp}\left(-t/\tau_{a}\right)$ in the TOF profiles, where $\tau_{a}$ is the absorption time, and therefore cancels in the intensity ratio $I_{\omega }\left(\rho ,t\right)/I_{\omega }\left(\rho =0,t\right)$ used to determine $w_{\rho }^{2}\left(t\right)$.

TOF data and the width squared in the diffuse regime were fitted using predictions of diffusion theory as described in (*24*). The source in the theory is a delta function in time, and, therefore, the predictions were convolved with the intensity envelope of the experimental input pulse, which is necessarily broadened in time due to the finite frequency bandwidth imposed by the filtering process. For the width squared, the only fitting parameter is the diffusion coefficient $D_{B}=\frac{1}{3}v_{E}\ell^{*}$, where $v_{E}$ is the energy velocity and $\ell^{*}$ is the transport mean free path, while the TOF profiles also involve the absorption time and amplitude coefficient.

The experimental data near the mobility edges were interpreted using a method involving the SCT of localization (*44*). In this model, the intensity Green’s function $C\left(\vec{r},\vec{r^{\prime }},\Omega \right)$ must satisfy both the diffusion equation

$$\left[-i\Omega -\nabla D\left(\vec{r},\Omega \right)\nabla \right]C\left(\vec{r},\vec{r^{\prime }},\Omega \right)=\delta \left(\vec{r}-\vec{r^{\prime }}\right)$$

and the renormalization condition involving the return probability

$$C\left(\vec{r},\vec{r},\Omega \right)$$

$$\frac{1}{D\left(\vec{r},\Omega \right)}=\frac{1}{D_{B}}+\frac{12\pi }{k^{2}l_{B}^{*}}C\left(\vec{r},\vec{r},\Omega \right)$$

Here, the Fourier transform of $C\left(\vec{r},\vec{r^{\prime }},\Omega \right)$, namely $C\left(\vec{r},\vec{r^{\prime }},t\right)$, describes the probability of finding a wave packet at a point $\vec{r}$ at a time *t* after a short pulse is emitted at $\vec{r^{\prime }}$, and the return probability $C\left(\vec{r},\vec{r},t\right)$ is simply the probability that the intensity returns to the same spot due to wave interference, thereby lowering the diffusion coefficient below the Boltzmann value. The fitting process used to compare the theoretical predictions of this model with the experimental data was performed frequency by frequency. Many of the input parameters for the code (both mean free paths $\ell_{s}$ and $\ell^{*}$, the wave number *k*, and the internal reflection coefficient *R*) were determined from the ballistic data, with both $\ell_{s}$ and *k* being measured directly. The transport mean free path was determined by taking into account the angular dependence of the scattering from a single scatterer and the ensuing relation between $\ell^{*}$ and $\ell_{s}$ [e.g., see (*35*)]; notably, at frequencies of interest near the quadrupolar resonance around 1.2 MHz, the difference between $\ell^{*}$ and $\ell_{s}$ is negligible (less than 1%) so that the numerical values of $\ell^{*}$ used in the code were taken to be equal to the experimental values of $\ell_{s}$ (which are in good agreement with predictions of the ISA in this frequency range). Note that the near equality of $\ell^{*}$ and $\ell_{s}$ implies that the scattering is isotropic on average, and this favors the enhancement of the return probability compared with the more usual situation of strong forward scattering ($\ell^{*}\gg \ell_{s}$). The reflection coefficient *R* was calculated as mentioned in (*24*) and described in detail for acoustic waves in (*53*). Hence, the only SCT fitting parameters were L/ξ or L/ζ, the diffusion time $\tau_{D}$ (which scales time in the theory), and, for the TOF profiles only, the absorption time $\tau_{a}$ and the TOF amplitude. For each frequency, we calculated the SCT for different L/ξ or/and L/ζ values and determined the best fit from the mininum in the reduced chi-squared

$$\chi_{\text{red}}^{2}=\frac{1}{N}\sum \frac{1}{\sigma_{i}^{2}}{\left[y_{\text{expt}}\left(t_{i}\right)-y_{\text{SCT}}\left(t_{i}\right)\right]}^{2}$$

Here, $\sigma_{i}$ corresponds to the experimental uncertainties at time $t_{i}$, N is the total number of fitting points, and y refers to either the width squared $w^{2}\left(t\right)$ or the TOF profile $I_{\omega }\left(\rho ,t\right)$, both of which were fitted simultaneously by finding the minimum in the average $\chi_{\text{red}}^{2}$ for these two quantities. In practice, taking as an example the most important fitting parameter L/ξ, its best (most probable) value is found by plotting $\chi_{\text{red}}^{2}$ versus L/ξ for a finite number of SCT calculations, fitting an inverted parabola to determine the value of L/ξ at the minimum, and its uncertainty $\sigma_{L/\xi }$ from the curvature via the relation

$${\sigma_{L/\xi }}^{2}=2{\left[\frac{\partial^{2}\chi_{\text{red}}^{2}}{\partial {\left(L/\xi \right)}^{2}}\right]}^{-1}$$

A similar approach in terms of ζ was used in the diffuse regime. Hence, the optimally fitted values of L/ξ and L/ζ were found for many frequencies near the first resonance of the beads where localization occurs.

### Statistics of intensity fluctuations in the speckle pattern

The measurements were conducted by depositing the samples directly on top of the piezoelectric element of a large rectangular transducer (active element, 50 mm by 80 mm; total surface area, 68 mm by 105 mm) to ensure an incident planar beam (Fig. 3A). The transducer was driven by a short broadband pulse consisting of a few cycles with a central frequency of 1.2 MHz. The cell supporting the sample was designed with a cross section larger than the transducer to minimize reflections from side walls. To further avoid any contamination of the signals from side-wall reflections, the transmitted fields were acquired over a grid of positions well away from the transducer edges using the hydrophone. The tip of the hydrophone was located in a thin layer of a pure gel (approximately millimeters) placed on the top surface of the sample to avoid mixing the beads while moving the hydrophone during signal acquisition.

The intensity I(x,y) for frequencies within the bandwidth of the incident pulse was obtained by taking the square of the FFT of δψ(x,y). Then, the averaged intensity ⟨I⟩ was obtained by averaging I(x,y) over the entire map for a given frequency, and the results were binned to determine P(I/⟨I⟩). Care was taken to ensure that the signal-to-noise ratio of the FFT magnitudes was sufficiently high that the ratio I/⟨I⟩ was not influenced by any noise background. To improve statistics, the results were averaged over a frequency band of 25 kHz, while still preserving adequate frequency resolution. The same experiments for ϕ = 20% were repeated four times on freshly prepared samples to measure the transmitted intensities for different configurations of the disorder, and the results were averaged in order to increase the reliability of the results.

The data were analyzed by first comparing the experimental results for P(I/⟨I⟩) with Rayleigh statistics, for which the contributions to the intensity from all multiply scattered paths through the sample are considered to be independent and uncorrelated (*46*). In this limit

P(I/⟨I⟩)=exp(−I/⟨I⟩)

However, in the approach to the localization regime, any method of analysis must account for interferences between the paths, which increase the probability of isolated high intensity peaks in the speckle pattern. Our method uses the NvR theory (*46*), which predicts that the probability distribution is given by the following double integral

$$P\left(I/\left\langle I\right\rangle \right)=\int_{0}^{\infty }\mathrm{dv}\frac{1}{v}\;\int_{-i\infty }^{i\infty }\mathrm{dx}\frac{1}{2\pi i}\text{exp}\left[-\frac{I/\left\langle I\right\rangle }{v}+\mathrm{xv}-\phi_{\text{con}}\left(x\right)\right]$$

with $\phi_{\text{con}}\left(x\right)=g\;{\text{ln}}^{2}\left[\sqrt{1+x/g}+\sqrt{x/g}\right].$ $\phi_{\text{con}}$ is the term that takes into account the multiple scattering interferences and is sensitive to the transducer beam (planar or Gaussian); it is given here for the planar case that is applicable to our data. This term also involves the dimensionless conductance g, which distinguishes between diffusive transport and localization (it is inversely proportional to the resistance for electrons). The predictions of the NvR theory were fitted to the experimental results to determine the most probable value of g for each frequency, using the same least-squares fitting procedure that was used for the transverse confinement data. To investigate the concentration dependence of the intensity statistics, the same experimental and theoretical approach was used to measure and interpret P(I/⟨I⟩) for a range of volume fractions ϕ of beads from 10 to 25%.
